# Phosphatidylinositol 4-kinase α suppresses glioblastoma progression by inactivating YAP and PI3K/Akt signaling

**DOI:** 10.1016/j.jbc.2026.113110

**Published:** 2026-05-06

**Authors:** Jinyuan Liu, Yiming Qian, Jing Zhang, Shuqiao Xing, Zhihui Huang, Yuanyuan Jiang

**Affiliations:** School of Pharmacy, Hangzhou Normal University, Hangzhou, Zhejiang, China

**Keywords:** glioblastoma multiforme, phosphatidylinositol 4-kinase α, PI3K/Akt, YAP

## Abstract

Glioblastoma multiforme (GBM) is one of the most malignant tumors of the central nervous system and is characterized by altered lipid metabolism. Notably, phosphatidylinositol (PI) metabolism is reprogrammed in GBM; however, its role and mechanism in GBM remain unclear. In this study, we found that phosphatidylinositol 4-kinase α (PI4Kα) (a subtype of PI4Ks) was downregulated in both low- and high-grade glioma tissues from clinical patients. Overexpressing the C terminus (1199–2102 amino acids) of PI4Kα, containing its catalytic domain (hereafter referred to as PI4Kα-CD for simplicity), in U251 and C6 cells (GBM cell lines), could significantly inhibit their proliferation and migration, whereas PI4Kα knockdown promoted their growth and migration. Mechanistically, PI4Kα inactivated YAP signaling by enhancing p-YAP (a major downstream effector of the Hippo pathway) and reducing the nuclear translocation of YAP, as well as suppressing PI3K/Akt signaling. YAP activation significantly restored the PI4Kα-CD overexpression-induced inhibitory effects on GBM growth. Finally, the growth of intracranially orthotopically transplanted PI4Kα-CD-overexpressing GBM cells in C57BL/6 mice was also suppressed through YAP signaling. Overall, these results reveal an unrecognized function of PI4Kα as a repressor in GBM progression through inactivation of YAP and PI3K/Akt signaling, thus providing a potential target for GBM treatment.

Gliomas are classified as grade I, II, III, and IV by the World Health Organization (1). Glioblastoma multiforme (GBM) belongs to grade IV and is the most fatal malignant brain tumor in adults with a poor prognosis (2, 3). Cellular metabolic reprogramming is one of the hallmarks of cancer (4). A recent study analyzing RNA-sequencing (RNA-seq) data from the Cancer Genome Atlas (TCGA) and Chinese Glioma Genome Atlas (CGGA) databases provided insight into the involvement of lipid metabolism in diffuse gliomas, and showed that glycosphingolipid metabolism is mainly enriched in GBM, whereas phosphatidylinositol (PI) metabolic progress is enriched in low-grade gliomas (LGGs) (5). Furthermore, metabolomics and lipidomics studies have shown that the PI content is relatively lower in glioma tissues than that in the paired adjacent tissues and negatively correlated with glioma malignancy (6), suggesting that PI metabolic reprogramming exists in gliomas.

PI can be phosphorylated by phosphatidylinositol kinases (PIKs) to generate phosphoinositides (PIPs), while phosphatidylinositol 4-kinases (PI4Ks) are enzymes that phosphorylate PI at the D-4 position of the hydroxyl group in the inositol ring to produce phosphatidylinositol 4-phosphate (PI4P), which is the most abundant PIP and widely distributed in various membrane components depending on the subcellular localizations and activities of PI4Ks (7). PI4P is a precursor for phosphatidylinositol 4, 5-bisphosphate [PI(4, 5)P2] and phosphatidylinositol 3, 4, 5-triphosphate [PI(3, 4, 5)P3] synthesis, as well as an important signaling molecule that plays crucial roles in maintaining organelle morphology, membrane vesicle transport, and lipid transport (8, 9, 10). Mammals have four PI4Ks, including type II isoforms PI4KIIα and PI4KIIβ, and type III isoforms PI4KIIIα (PI4Kα) and PI4KIIIβ (PI4Kβ) (11). The roles and mechanisms of PI4Ks have been explored in some tumor types. PI4Kα contributes to the undifferentiated status and poor prognosis in human hepatocellular carcinoma (12). In prostate cancer, PI4Kα overexpression promotes a more metastatic phenotype depending on its crosstalk with CXCR4 (13). Blocking PI4Kα-mediated PI4P synthesis in the plasma membrane inhibits the growth of KRAS-driven cancers *via* suppression of ORP5 and ORP8 function (14). Besides, PI4Kβ is a novel driver of breast cancer (15, 16, 17) and a therapeutic target in lung adenocarcinoma (18). Targeting PI4K2α/PIR lysosome network will be a potential strategy for cancer treatment (19). However, the roles and mechanisms of PI4Ks in GBM progression remain to be explored.

In the present study, we found that *PI4Kα* transcription was significantly different in glioma tissues compared with that in the normal brain tissues, and showed a negative correlation between *PI4Kα* expression and glioma malignancy in data from public databases. Based on this, we found that overexpression of PI4Kα-CD suppressed, whereas knockdown of PI4Kα enhanced the proliferation and migration of GBM cells, respectively. Mechanistically, PI4Kα inactivated YAP and PI3K/Akt signaling. Overall, these results provide evidence that PI4Kα acts as a GBM suppressor by regulating YAP and PI3K/Akt signaling as well as a potential prognostic biomarker and drug target for GBM treatment.

## Results

### PI4Kα was downregulated, negatively correlated with the glioma malignancy, and widely expressed in GBM cell lines

**Figure 1:**
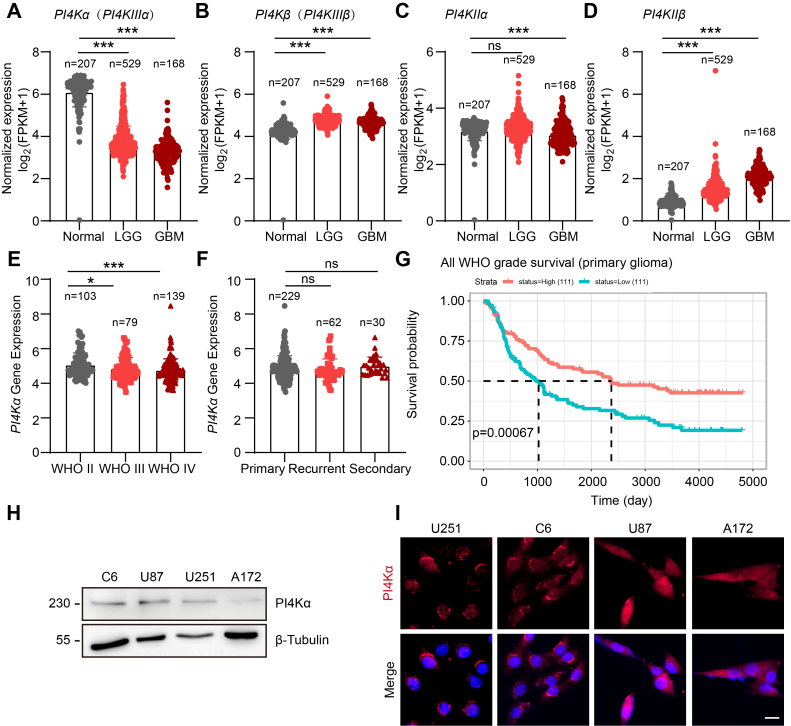
**PI4Kα was downregulated, negatively correlated with the glioma malignancy and widely expressed in GBM cell lines.***A*–*D*, the transcriptional expression of *PI4Kα* (*A*), *PI4Kβ* (*B*), *PI4KIIα* (*C*), and *PI4KIIβ* (*D*) in both LGG and GBM tissues from clinical patients compared to that in normal tissues from the TCGA and GTEx databases (normal, n = 207; LGG, n = 529; GBM, n = 168, Kruskal-Wallis test, ∗∗∗*p* < 0.001). *E*, the *PI4Kα* gene expression in WHO II, III and IV glioma tissues from the CGGA database (WHO II, n = 103; WHO III, n = 79; WHO IV, n = 139, one-way ANOVA, ∗*p* < 0.05, ∗∗∗*p* < 0.001). *F*, the *PI4Kα* gene expression in primary, recurrent, and secondary glioma tissues from CGGA database (Primary, n = 229; Recurrent, n = 62; Secondary, n = 30, one-way ANOVA). *G*, the survival probability of primary glioma patients with high or low expression of *PI4Kα* (log-rank test). *H* and *I*, Western blotting (*H*) and immunofluorescence staining (*I*) were used to detect the PI4Kα expression in GBM cell lines, including C6, U87, U251, and A172 cells. Scale bar: 20 μm. Data were represented as mean ± SD.

To investigate the potential role of PI4Ks in GBM progression, we first analyzed the gene expression of PI4K isoforms, *PI4Kα*, *PI4Kβ*, *PI4KIIα*, and *PI4KIIβ*, in glioma tissues compared with that in the normal brain tissues based on the TCGA and GTEx (Genotype-Tissue Expression) databases. As shown in Figure 1, *A*–*D*, *PI4Kα* showed a statistically decreased expression level in both LGG and GBM than in normal tissues. Further, analysis of the CGGA database showed that lower *PI4Kα* expression was correlated with a higher grade of glioma (Fig. 1*E*). However, *PI4Kα* expression was not significantly changed among the primary, recurrent, and secondary glioma tissues (Fig. 1*F*). Besides, survival probability analysis of the CGGA database showed that lower *PI4Kα* expression was also associated with a shorter survival of patients with primary glioma (Fig. 1*G*). Furthermore, we also found that PI4Kα was widely expressed in GBM cells, including C6 rat GBM cells and U87, U251, and A172 human GBM cells (Fig. 1, *H* and *I*). Collectively, these results suggested that PI4Kα was downregulated, negatively correlated with glioma malignancy, and widely expressed in GBM cell lines, indicating that PI4Kα expression may serve as a bio-diagnostic marker to predict the prognosis of patients with glioma.

### PI4Kα inhibited the viability, proliferation, and migration of GBM cells *in vitro*

**Figure 2:**
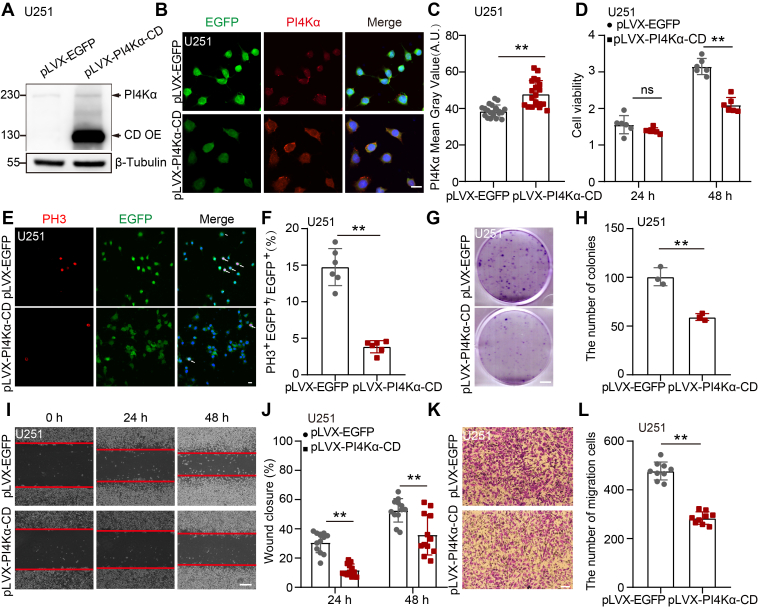
**Overexpression of PI4Kα inhibited the viability, proliferation, and migration of U251 cells *in vitro*.***A*, Western blotting detected the expression of PI4Kα in U251 cells: the upper arrow indicated the endogenous full-length PI4Kα, and the lower arrow indicated the overexpressed PI4Kα-CD-EGFP. *B*, representative image of immunofluorescence staining of PI4Kα in control and PI4Kα-CD-overexpressing U251 cells. Scale bar: 20 μm. *C*, quantitative analysis of PI4Kα expression as shown in (*B*) (n = 20, *t* test, ∗∗*p* < 0.01). *D*, CCK-8 assay detected the cell viability of control and PI4Kα-CD-overexpressing U251 cells (n = 6, two-way ANOVA, ∗∗*p* < 0.01). *E*, representative image of PH3 staining in control and PI4Kα-CD-overexpressing U251 cells. Scale bar: 20 μm. *F*, quantitative analysis of the percentages of PH3^+^EGFP^+^/EGFP^+^ cells as shown in (*E*) (n = 6, *t* test, ∗∗*p* < 0.01). *G*, cell colony formation assay detected the proliferation ability of control and PI4Kα-CD-overexpressing U251 cells. Scale bar: 5 mm. *H*, quantitative analysis of the number of cell colonies as shown in (*G*) (n = 3, *t* test, ∗∗*p* < 0.01). *I*, Wound healing assay detected the migration of control and PI4Kα-CD-overexpressing U251 cells at 24 h and 48 h after scratch. Scale bar: 200 μm. *J*, quantitative analysis of the percentage of wound closure as shown in (*I*) (n = 12, two-way ANOVA, ∗∗*p* < 0.01). *K*, Transwell assay detected the migration ability of control and PI4Kα-CD-overexpressing U251 cells. Scale bar: 50 μm. *L*, quantitative analysis of the number of migrated cells as shown in (*K*) (n = 9, *t* test, ∗∗*p* < 0.01). Data were represented as mean ± SD.

To further examine the role of PI4Kα in GBM progression, a PI4Kα-CD-overexpression plasmid, pLVX-CMV-PI4Kα-CD-EGFP-IRES-Neo (pLVX-PI4Kα-CD), was constructed *via* molecular cloning. Both pLVX-PI4Kα-CD and the control plasmid, pLVX-CMV-EGFP-IRES-Neo (pLVX-EGFP), were subjected to lentiviral packaging for GBM cell transfection. As shown in Figures 2*A* and S1*A*, the overexpressed PI4Kα-CD-EGFP protein band was observed around the 130 kD position, whereas the full-length endogenous PI4Kα was observed at 230 kD in both U251-PI4Kα-CD and C6-PI4Kα-CD cells. Immunostaining further confirmed the PI4Kα overexpression in transfected EGFP^+^ GBM cells (Figs. 2, *B* and *C* and S1, *B* and *C*). Next, the results of the Cell Counting Kit-8 (CCK-8) assay (a method to detect cell viability) showed that the cell viability was significantly inhibited in both U251- and C6-PI4Kα-CD cells (Figs. 2*D* and S1*D*). Subsequently, immunostaining of PH3 (a marker of cell proliferation) showed that the percentages of PH3^+^EGFP^+^/EGFP^+^ cells were significantly decreased in both U251- and C6-PI4Kα-CD cells (Figs. 2, *E* and *F* and S1, *E* and *F*). Further, the cell colony formation assay showed that the number of cell colonies was reduced in both U251- and C6-PI4Kα-CD cells (Figs. 2, *G* and *H* and S1, *G* and *H*). Taken together, these results suggested that PI4Kα-CD overexpression inhibited the viability and proliferation of GBM cells.

To further examine the effects of PI4Kα on GBM cell migration, we first performed a wound healing assay. As shown in Figures 2, *I* and *J* and S1, *I* and *J*, the percentages of wound closure were significantly reduced in both U251- and C6-PI4Kα-CD cells at both 24 h and 48 h. Furthermore, the Transwell migration assay results showed markedly decreased numbers of migrated cells for both U251 and C6-PI4Kα-CD cells (Figs. 2, *K* and *L* and S1, *K* and *L*). Together, these results strongly suggested that PI4Kα inhibited GBM cell migration *in vitro*.

**Figure 3:**
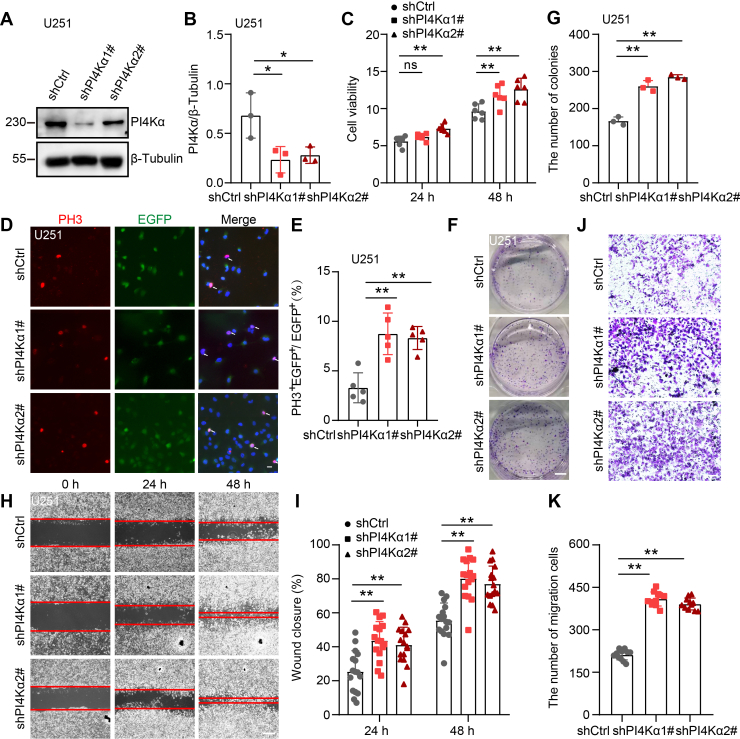
**Knockdown of PI4Kα promoted the viability, proliferation, and migration of U251 cells *in vitro*.***A*, Western blotting detected the expression of PI4Kα in U251-shCtrl, U251-shPI4Kα1#, and U251-shPI4Kα2# cells. *B*, quantitative analysis of the relative PI4Kα expression as shown in (*A*) (n = 3, one-way ANOVA, ∗*p* < 0.05). *C*, CCK-8 assay detected the viability of U251-shCtrl, U251-shPI4Kα1#, and U251-shPI4Kα2# cells (n = 6, two-way ANOVA, ∗∗*p* < 0.01). *D*, immunofluorescence staining of PH3 in U251-shCtrl, U251-shPI4Kα1#, and U251-shPI4Kα2# cells. Scale bar: 20 μm. *E*, statistical analysis of the percentage of PH3^+^EGFP^+^/EGFP^+^ cells as shown in (*D*) (n = 5, one-way ANOVA, ∗∗*p* < 0.01). *F*, representative images showing the cell colony formation of U251-shCtrl, U251-shPI4Kα1#, and U251-shPI4Kα2# cells. Scale bar: 5 mm. *G*, quantitative analysis of colony number as shown in (*F*) (n = 3, one-way ANOVA, ∗∗*p* < 0.01). *H*, wound healing assay detected the migration of U251-shCtrl, U251-shPI4Kα1#, and U251-shPI4Kα2# cells at 24 h and 48 h after scratch. Scale bar: 200 μm. *I*, quantitative analysis of the percentage of wound closure as shown in (*H*) (n = 16, two-way ANOVA, ∗∗*p* < 0.01). *J*, Transwell assay detected the migration ability of U251-shCtrl, U251-shPI4Kα1#, and U251-shPI4Kα2# cells. Scale bar: 50 μm. *K*, quantitative analysis of the number of migrated cells as shown in (*J*) (n = 11, one-way ANOVA, ∗∗*p* < 0.01). Data were represented as mean ± SD.

In addition, we established lentiviral shRNA-mediated PI4Kα-knockdown in U251 cells (Fig. 3, *A* and *B*). As expected, the cell viability was significantly enhanced in U251-shPI4Kα1# and U251-shPI4Kα2# cells as determined using the CCK-8 assay (Fig. 3*C*); further, the proliferation ability, examined by PH3 immunostaining (Fig. 3, *D* and *E*) and cell colony formation assay, was also increased (Fig. 3, *F* and *G*). PI4Kα knockdown significantly promoted wound healing in U251 cells (Fig. 3, *H* and *I*), and increased the number of migrated U251-shPI4Kα1# and U251-shPI4Kα2# cells in transwell assay (Fig. 3, *J* and *K*). In conclusion, these results suggested that PI4Kα knockdown promoted the viability, proliferation, and migration of GBM cells.

### PI4Kα regulated the YAP and PI3K/Akt signaling pathways in GBM cells

Hippo/YAP has been reported to modulate cell growth, differentiation, metabolism, and tumorigenesis (20, 21, 22). A previous study has defined pan-cancer binary classes as YAP^on^ (pro-cancer YAP activity) or YAP^off^ (anti-cancer YAP activity) types (23), and GBM is a YAP^on^ tumor. Recently, PI4Kα-mediated PI4P synthesis in the plasma membrane was reported to be involved in modulating the Hippo/YAP pathway (24). Based on this rationale, we asked whether PI4Kα regulated GBM progression *via* modulating YAP signaling.

**Figure 4:**
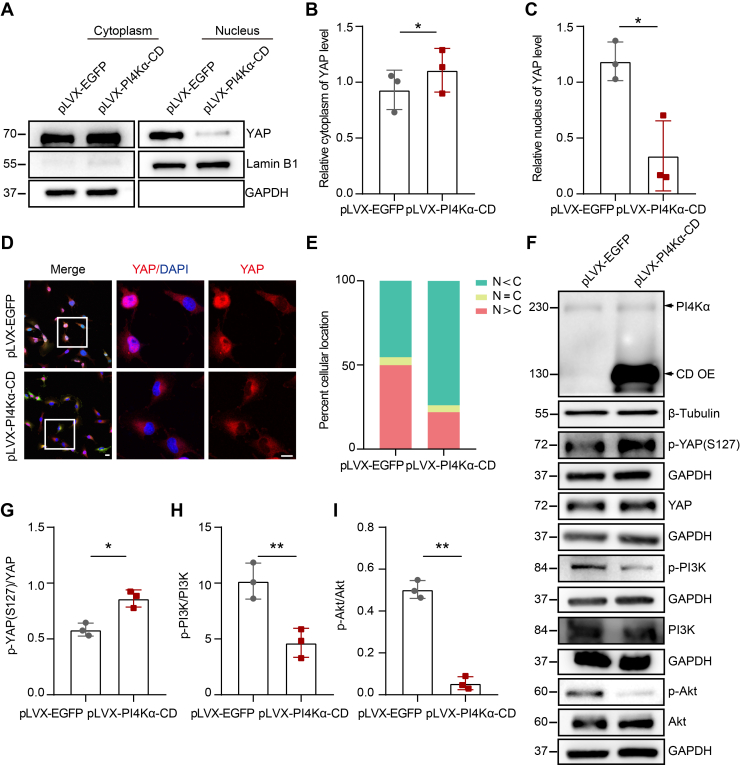
**PI4Kα overexpression inactivated YAP and PI3K/Akt signaling pathways in GBM cells.***A*, Western blotting was performed to detect the expression of cytosolic and nuclear YAP in control and PI4Kα-CD-overexpressing U251 cells. *B* and *C*, quantitative analysis of the relative expression of YAP in cytoplasm (*B*) and nuclear (*C*) as shown in (*A*) (n = 3, *t* test, ∗*p* < 0.05). *D*, representative image of YAP immunofluorescence staining (*Red*) in control and PI4Kα-CD-overexpressing U251 cells, Scale bar: 20 μm. *E*, quantification of YAP subcellular localization: EGFP^+^ cells were quantified based on YAP localization signals: N > C: more nuclear YAP, N < C: more cytosolic YAP, N = C: nuclear YAP and cytosolic YAP behave similar (about 100 cells in randomly selected fields were counted). (*F*) Western blotting detected the expression of p-YAP/YAP, p-PI3K/PI3K, and p-Akt/Akt in control and PI4Kα-CD-overexpressing U251 cells. *G–I*, quantitative analysis of the relative expression of proteins detected as shown in (*F*) (n = 3, *t* test, ∗*p* < 0.05, ∗∗*p* < 0.01). Data were represented as mean ± SD.

First, by performing cellular nuclear cytoplasmic separation followed by western blotting, we examined whether PI4Kα regulated the sub-cellular distribution of YAP as YAP is a transcriptional co-activator. The results showed that PI4Kα-CD overexpression significantly suppressed the nuclear translocation of YAP but promoted the cytosolic translocation of YAP (Fig. 4, *A*–*C*). Moreover, YAP immunostaining showed that the percentage of cells with nuclear YAP localization was indeed significantly reduced in PI4Kα-CD-overexpressing U251 cells (Fig. 4, *D* and *E*). Furthermore, western blotting showed that the protein level of p-YAP (S127)/YAP was indeed increased in U251-PI4Kα-CD cells (Fig. 4, *F* and *G*), suggesting that PI4Kα inactivated YAP. PI signaling is involved in various intracellular signaling pathways (25, 26); further, the PI3K/Akt signaling pathway is well-known in regulating cancer progression. Previous studies have reported that YAP and PI3K/Akt are closely related to the regulation of tumor progression (27, 28, 29). Notably, the expression of p-PI3K/PI3K and p-Akt/Akt was also down-regulated in U251-PI4Kα-CD cells (Fig. 4, *F* and *H* and *I*). Taken together, these results suggested that the overexpression of PI4Kα-CD inactivated the YAP and PI3K/Akt pathways.

**Figure 5:**
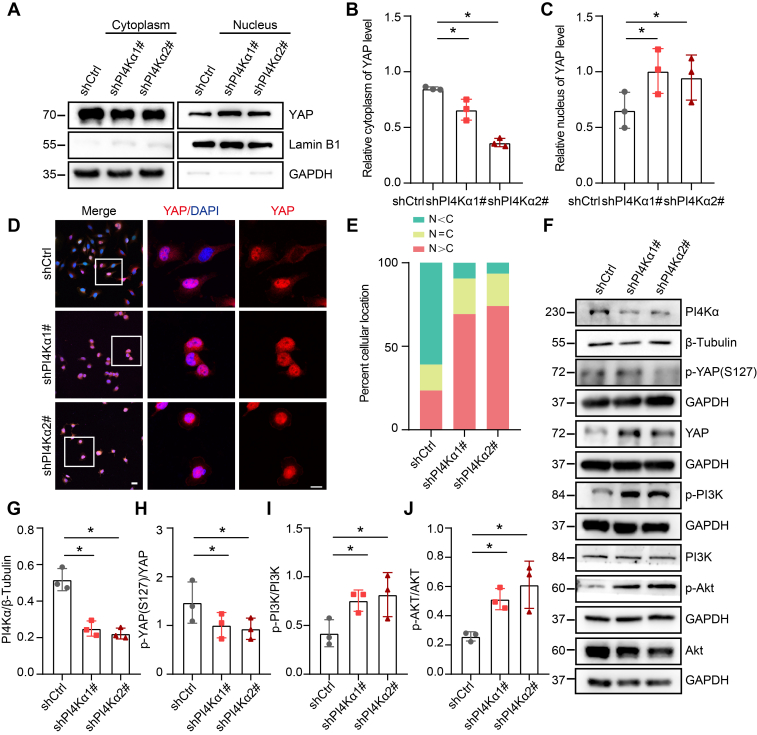
**Knockdown of PI4Kα activated YAP and PI3K/Akt signaling in GBM cells.***A*, Western blotting detected the expression of cytosolic and nuclear YAP in U251-shCtrl and U251-shPI4Kα cells, respectively. *B* and *C*, quantitative analysis of the relative expression of YAP in cytoplasm (*B*) and nuclear (*C*) as shown in (*A*) (n = 3, one-way ANOVA, ∗*p* < 0.05). *D*, representative image of YAP immunofluorescence staining (*Red*) in U251-shCtrl and U251-shPI4Kα cells, Scale bar: 20 μm. *E*, quantification of YAP subcellular localization as shown in (*D*). *F*, Western blotting detected the expression of p-YAP/YAP, p-PI3K/PI3K, and p-Akt/Akt in U251-shCtrl and U251-shPI4Kα cells. *G–J*, quantitative analysis of the relative expression of proteins detected as shown in (*F*) (n = 3, one-way ANOVA, ∗*p* < 0.05). Data were represented as mean ± SD.

Meanwhile, knockdown of PI4Kα promoted the nuclear translocation of YAP (Fig. 5, *A*–*E*). Furthermore, the protein level of p-YAP (S127)/YAP was reduced (Fig. 5, *F*–*H*), whereas p-PI3K/PI3K and p-Akt/Akt were up-regulated as expected (Fig. 5, *F*, *I*, and *J*). Taken together, these results suggested that PI4Kα inactivated the YAP and PI3K/Akt pathways in GBM cells.

### PI4Kα inhibited GBM cell growth by inactivating the YAP and PI3K/Akt signaling pathways

**Figure 6:**
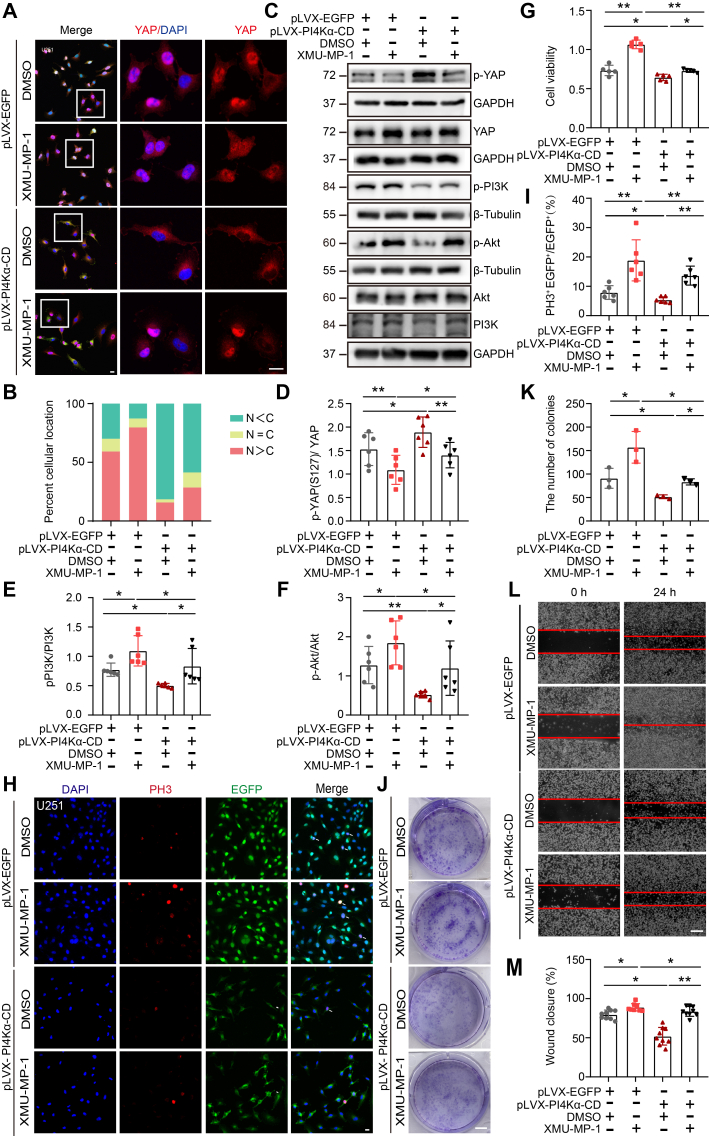
**Activation of YAP by XMU-MP-1 restored the inhibitory effects on GBM cells caused by PI4Kα overexpression *via* PI3K/Akt signaling.***A*, representative image of YAP immunofluorescence staining (*Red*) in U251-control and PI4Kα-CD-overexpressing cells with or without XMU-MP-1 treatment. EGFP represents the transfected cells, Scale bar: 20 μm. *B*, quantification of YAP subcellular localization as shown in (*A*). *C*, Western blotting detected the expression of p-YAP/YAP, p-PI3K/PI3K, and p-Akt/Akt in U251-control and PI4Kα-CD-overexpressing cells with or without XMU-MP-1 treatment. *D–F*, quantitative analysis of the relative expression of proteins detected as shown in (*C*) (n = 6, one-way ANOVA, ∗*p* < 0.05, ∗∗*p* < 0.01). *G*, CCK-8 assay measured the viability of U251-control and PI4Kα-CD overexpression cells with or without XMU-MP-1 treatment (n = 5, one-way ANOVA, ∗*p* < 0.05, ∗∗*p* < 0.01). *H*, representative image of PH3 immunofluorescence staining (*red*) in U251-control and PI4Kα-CD-overexpressing cells with or without XMU-MP-1 treatment. EGFP represents the transfected cells. Scale bar: 20 μm. *I*, quantification of the percentages of PH3^+^EGFP^+^/EGFP^+^ cells as shown in (*H*) (n = 6, one-way ANOVA, ∗*p* < 0.05, ∗∗*p* < 0.01). *J*, Representative image of the formation of cell colonies in U251-control and PI4Kα-CD-overexpressing cells with or without XMU-MP-1 treatment. Scale bar: 5 mm. *K*, quantification of the number of cell colonies as shown in (*J*) (n = 3, one-way ANOVA, ∗*p* < 0.05). *L*, wound healing assay detected the migration ability of U251-control and PI4Kα-CD-overexpressing cells with or without XMU-MP-1 treatment. Scale bar: 200 μm. *M*, quantification of the percentages of wound healing rate as shown in (*L*) (n = 10, one-way ANOVA, ∗*p* < 0.05, ∗∗*p* < 0.01). Data were represented as mean ± SD.

To determine whether PI4Kα inhibited GBM growth by inactivating YAP and PI3K/Akt signaling, XMU-MP-1, an inhibitor of the Hippo signaling pathway, was used to activate YAP (30) in the rescue experiment. Indeed, the CCK-8 assay showed that XMU-MP-1 significantly promoted the viability of U251 cells (Fig. S2). Meanwhile, XMU-MP-1 promoted the nuclear translocation of YAP in U251-control cells and restored the reduced percentage of cells with nuclear YAP caused by PI4Kα-CD overexpression (Fig. 6, *A* and *B*), along with restoring the upregulated expression of p-YAP (S127)/YAP (Fig. 6, *C* and *D*). Further, p-PI3K/PI3K and p-Akt/Akt were upregulated upon XMU-MP-1 treatment in control cells and their downregulation induced by PI4Kα-CD overexpression was also rescued by XMU-MP-1 (Fig. 6, *C*, *E*, and *F*). The CCK-8, PH3 immunostaining, and cell-colony formation assays showed that XMU-MP-1 enhanced the viability and proliferation of control cells and restored the inhibitory effects of PI4Kα-CD overexpression on U251 cells (Fig. 6, *G*–*K*). Furthermore, XMU-MP-1 also enhanced U251 cell migration and restored the inhibitory effects on cell migration in U251-PI4Kα-CD cells (Fig. 6, *L* and *M*). These results suggested that PI4Kα inhibited GBM cell growth by inactivating the YAP and PI3K/Akt signaling pathways.

### PI4Kα impeded GBM progression *in vivo*

**Figure 7:**
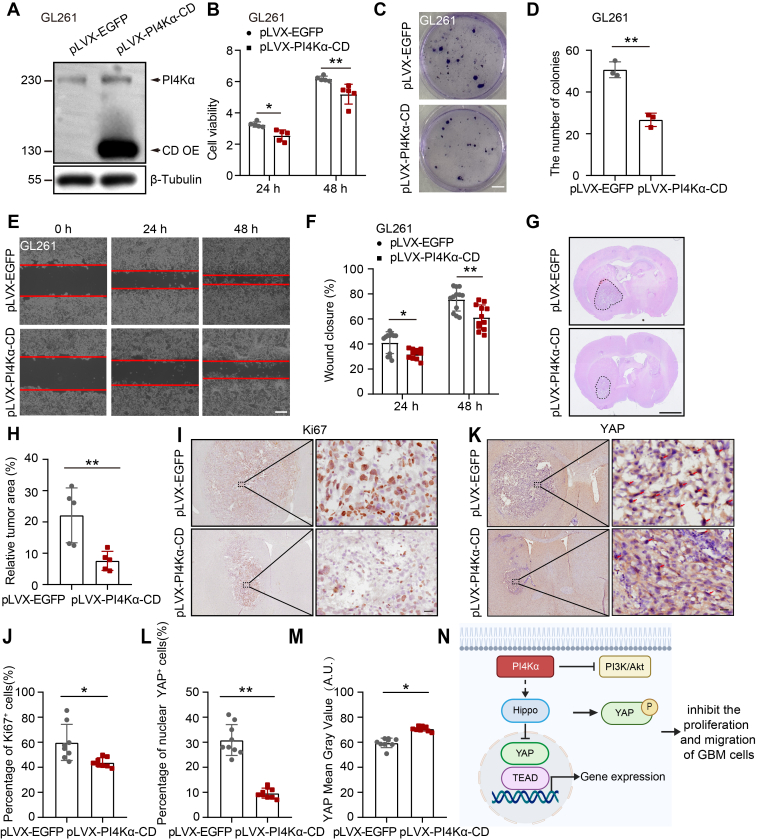
**PI4Kα overexpression inhibited GL261 proliferation and migration *in vitro* and inhibited GBM growth *via* inactivating YAP *in vivo*.***A*, Western blotting detected the overexpression of PI4Kα in GL261 cells: the *upper arrow* and the *lower arrow* indicated the endogenous full-length PI4Kα and the overexpressed PI4Kα-CD, respectively. *B*, CCK-8 assay measured the cell viability of control and PI4Kα-CD-overexpressing GL261 cells (n = 6, two-way ANOVA, ∗*p* < 0.05, ∗∗*p* < 0.01). *C*, cell colony formation assay was performed in GL261-control and GL261-PI4Kα-CD-overexpressing cells. Scale bar: 5 mm. *D*, quantitative analysis of the number of cell colonies as shown in (*C*) (n = 3, *t* test, ∗∗*p* < 0.01). *E*, wound healing assay detected the migration of GL261 cells with or without PI4Kα overexpression at 24 h and 48 h after scratch. Scale bar: 200 μm. *F*, quantitative analysis of the percentage of wound closure as shown in (*E*) (n = 12, two-way ANOVA, ∗*p* < 0.05, ∗∗*p* < 0.01). *G*, representative image of HE staining of frozen brain sections from GBM-bearing mice *via* transplanting GL261-control or GL261-PI4Kα-CD cells, respectively. Scale bar: 2 mm. *H*, quantitative analysis of the relative tumor area as shown in (*G*) (n = 5, *t* test, ∗∗*p* < 0.01). *I*, representative image of immunohistochemical staining of Ki67 to detect the proliferation of GL261-control and GL261-PI4Kα-CD transplanted mice. Scale bar: 20 μm. *J*, quantitative analysis of the percentage of Ki67^+^ cells as shown in (*I*) (n = 8 sections, *t* test, ∗*p* < 0.05). *K*, representative image of immunohistochemical staining of YAP in GBM tumors from GL261-control and GL261-PI4Kα-CD transplanted mice. Scale bar: 20 μm. *L*, quantitative analysis of the percentages of nuclear YAP^+^ cells as shown in (*K*) (n = 9 sections, *t* test, ∗∗*p* < 0.01). *M*, quantitative analysis of the mean gray value of YAP as shown in (*L*) (n = 9 sections, *t* test, ∗*p* < 0.05). *N*, graphic abstract: PI4Kα inactivated YAP and PI3K/Akt, thus suppressing the proliferation and migration of GBM cells. Data were represented as mean ± SD.

To investigate whether PI4Kα also suppressed intracranial GBM progression *in vivo*, we first overexpressed PI4Kα-CD in the murine GBM cell line GL261 *via* lentivirus transfection, as described above (Fig. 7*A*). The CCK-8, cell colony formation, and wound-healing assays confirmed the inhibitory effect on GL261 proliferation and migration *in vitro* upon PI4Kα-CD overexpression (Fig. 7, *B*–*F*). The control and GL261-PI4Kα-CD cells were then transplanted into the striatum of C57BL/6 mice, respectively. As shown in Figure 7, *G* and *H*, orthotopic GBM growth was significantly inhibited by PI4Kα-CD overexpression. Ki67 immunohistochemical staining showed decreased proliferation in PI4Kα-CD-overexpressing GL261 xenograft tumors (Fig. 7, *I* and *J*). To further determine whether PI4Kα impeded GBM progression by inactivating YAP as found *in vitro*, immunohistochemical staining using an anti-YAP antibody was performed on brain frozen sections from the control and PI4Kα-CD-overexpressing GL261 transplanted groups. The overall YAP expression was slightly elevated but the activated nuclear YAP was notably decreased as expected (Fig. 7, *K*–*M*). These results suggested that PI4Kα suppressed GBM progression *in vivo* by inactivating YAP signaling, which was consistent with our *in vitro* results.

## Discussion

In this study, we found that *PI4Kα* expression was downregulated in glioma tissues and negatively correlated with glioma malignancy. Furthermore, we revealed that PI4Kα inactivated YAP and PI3K/Akt signaling, thus suppressing GBM progression both *in vitro* and *in vivo* (Fig. 7*N*). Our results suggest that PI4Kα acts as a GBM suppressor, thus providing a potential prognostic biomarker for gliomas as well as a target for GBM treatment.

Lipid metabolism may play an important role in human cancer development by affecting various cellular processes, including cell growth, proliferation, differentiation, and motility (31, 32, 33). Transcriptomic, metabolomic, and lipidomic analyses indicated that gliomas underwent a reprogramming of PI metabolism (5, 6). The inositol ring of PI has three hydroxyl group sites that can be phosphorylated by kinases to produce seven types of PIPs, including three monophosphorylated PIs (PI3P, PI4P, PI5P) and three bisphosphorylated PIs (PI(3, 4)P2, PI(3, 5)P2, PI(4, 5)P2), and one triphosphorylated PI (PI(3, 4, 5)P3) (34). Tumor-related gene mutations can lead to the elevation of PIPs; for example, PI(4, 5)P2 and PI(3, 4, 5)P3 are increased because of mutations in PIP kinase, PI3K, and/or PTEN, which play an important role in tumor progression (35, 36). Mammals have four PI4Ks, including PI4KIIα, PI4KIIβ, PI4Kα, and PI4Kβ, which catalyze PI into PI4P. Several studies have reported the role of PI4Ks in tumor progression (12, 13, 14, 15, 16, 17, 18, 19); however, few studies have examined their roles in glioma.

Therefore, we first analyzed the transcriptional information of PI4Ks in the open database, and found that, among the four PI4K isoforms, *PI4Kα* was downregulated in glioma tissues from patients compared with that in normal brain tissues, with higher grades of glioma showing lower *PI4Kα* expression. Furthermore, PI4Kα was widely expressed in GBM cells. Subsequently, *in vitro* experiments showed that the viability, proliferation, and migration of GBM cells were significantly suppressed by lentivirus-mediated PI4Kα overexpression, whereas lentivirus-mediated shPI4Kα accelerated GBM cell growth. Therefore, these results suggest that PI4Kα acted as a GBM suppressor.

GBM is a YAP^on^ tumor. YAP is a core downstream effector of the Hippo pathway, and when Hippo is activated, activated MST1/2 subsequently phosphorylates and activates SAV1, LATS1/2, and MOB1, leading to the phosphorylation and retention of YAP/TAZ in the cytoplasm for degradation; in contrast, when Hippo becomes inactive, non-phosphorylated YAP enters the nucleus and binds TEADs to initiate the transcription of downstream target genes (37, 38, 39). Studies have also revealed a correlation between PI signaling and the Hippo pathway. Examples include osmotic stress-stimulated Hippo signaling *via* PI5K (40). Further, recent studies have shown PI transfer protein (PITP) as a new regulator for the Hippo/YAP pathway, in which process, PITP transfers PI and facilitates PI4Kα-mediated plasma membrane PI4P synthesis, mediating NF2 (which contains a PIP-binding domain) inhibition, and thus, Hippo inactivation and YAP activation (24). Moreover, PI3K/Akt is a well-known PI signaling pathway that first became a focus in the cancer field in the mid-1980s (41). Overall, Hippo/YAP and PI3K/Akt are closely related to the modulation of tumor progression (27, 28, 29).

Consistent with these previous studies, we also found that PI4Kα modulated YAP in GBM. PI4Kα-CD overexpression significantly suppressed YAP nuclear translocation and upregulated cytosolic YAP expression, whereas PI4Kα knockdown promoted YAP nuclear translocation. Further, the expression of p-YAP (S127)/YAP was increased in PI4Kα-CD-overexpressing GBM cells, whereas PI4Kα knockdown induced the opposite effect. Further, p-PI3K/PI3K and p-Akt/Akt were also down-regulated in PI4Kα-CD-overexpressing GBM cells, but upregulated in PI4Kα-knockdown cells. Taken together, these results suggested that PI4Kα inactivated YAP and PI3K/Akt, which may contribute to its inhibitory role in GBM growth. To further confirm this hypothesis, we used XMU-MP-1, a YAP activator, and we found that YAP activation by XMU-MP-1 restored the inhibitory effect on GBM cell growth caused by PI4Kα-CD overexpression, along with activating PI3K/Akt signaling. Consistent with the *in vitro* results, overexpression of PI4Kα inhibited GBM progression *via* YAP signaling *in vivo*. These results strongly suggested that PI4Kα inhibited GBM progression *via* YAP and PI3K/Akt signaling both *in vitro* and *in vivo*. Besides, XMU-MP-1 could only partially rescue the inhibitory effect caused by PI4Kα-CD in GBM cells, suggesting that YAP is not a unique downstream effector of PI4Kα.

PI4Kα is known to be an essential lipid kinase that produces the predominant pool of PI4P at the plasma membrane, and PI4Kα activation is associated with multiple regulatory proteins, including TTC7, FAM126, and EFR3, whereas dysregulation of PI4Kα or its regulatory protein is associated with multiple human diseases, including cancer (42). PI4Kα directly interacts with TTC7, whereas FAM126 does not bind directly with PI4Kα but a stabilizer of TTC7; EFR3 interacts directly with both TTC7 and FAM126 to achieve the plasma membrane localization of PI4Kα (43, 44, 45). Human PI4Kα (2102 amino acids) is a multi-domain protein, including an N-terminal α-Solenoid domain (1–956 residues), a dimerization domain (957–1536 residues), a cradle domain (1537–1787 residues), and a catalytic domain (1788–2085 residues), and deletion of 1134 amino acids of the PI4Kα N-terminus retains its interaction with the TTC7/FAM126 complex (43). Our shRNA experiments support the PI4Kα-CD overexpression results in GBM even though 1198 amino acids of the PI4Kα N-terminus were removed, suggesting that PI4Kα-CD (1199–2102 amino acids of PI4Kα comprising most of the region of the dimerization domain, the cradle domain, and the catalytic domain) is still functional. However, efforts are needed to obtain the full-length of PI4Kα.

Notably, PI4K activity is believed to enhance PI4P synthesis, which will generate more PI(4, 5)P2 and PI(3, 4, 5)P3, thus promoting cell growth. However, unlike treatment with PI4Kα inhibitor GSK-A1 could induce cytoplasmic YAP translocation, and overexpression of PI4Kα induced activated nuclear YAP localization in HEK293A cells (24). In this study, PI4Kα inactivated YAP and suppressed GBM cell growth, whereas PI4Kα knockdown promoted nuclear YAP translocation to enhance GBM progression. Therefore, we speculated that in GBM cells, PI4P production was not increased, but was rather decreased upon PI4Kα overexpression, which possibly because of compensatory regulation by other subtypes of PI4Ks or others, which we will examine in future studies.

In summary, our studies provide evidence that PI4Kα acts as a GBM suppressor by regulating YAP and PI3K/Akt signaling as well as a potential prognostic biomarker and drug target for GBM.

## Experimental procedures

### Expression level analysis

The gene expression of *PI4Ks* in LGG and GBM tissues from the clinic was collected from the TCGA database, whereas their expression in normal brain tissues was collected from GTEx database, and these RNA-seq data were performed Log_2_(FPKM+1) transformation, thus the differential expression analysis was analyzed. Additionally, the CGGA database was also used to analyze the *PI4Kα* gene expression across different WHO glioma grades, as well as in primary, recurrent, and secondary glioma tissues. The correlation of *PI4Kα* expression and patient survival was also evaluated using the CGGA database (46).

### Cell lines and animals

The rat GBM cell line C6 and human GBM cell line U251 were kindly gifted from Prof. Maojin Yao (Sun Yat-Sen University), while the murine GBM cell line GL261 was purchased from Tongpai Biotechnology Co, Ltd. The HEK-293T cell line was provided by Prof. Ying Wang (Affiliated Hangzhou First People's Hospital). All cells were cultured in DMEM supplemented with 10% fetal bovine serum (FBS) and 1% penicillin/streptomycin in an atmosphere with 5% CO_2_ at 37 °C.

Male C57BL/6 mice (6–8 weeks old; 18–22*g* weight) were purchased from Shanghai SLAC Laboratory Animal Co, Ltd. All animal experiments were performed according to the protocols approved by Animal Care and Use Committee of Hangzhou Normal University (HSD20230503).

### Plasmid construction

For PI4Kα overexpression, 1199 to 2102 amino acids of human PI4Kα cDNA (the full-length of PI4Kα is 2102 amino acids) that contains its catalytic domain which we described as PI4Kα-CD was amplified and inserted into pEGFP-N2 using restriction enzyme sites HindIII and EcoRI *via* ligase-independent cloning. The primer sequences for PI4Kα-CD amplification were as follows: the forward primer: 5′-TCTCGAGCTCAAGCTTGCCACCATGCTCATTAGCAGTAAAGATT-3′; the reverse primer: 5′-GTCGACTGCAGAATTCGTAGGGGATGTCATTCTGATAG-3′. The constructed plasmid pEGFP-N2-PI4Kα-CD was verified by sequencing. Subsequently, restriction enzymes XhoI and NotI were used to obtain the EGFP from pEGFP-N2 and PI4Kα-CD-EGFP from pEGFP-N2-PI4Kα-CD, and then inserted into the lentiviral vector pLVX-CMV-IRES-Neo, respectively. The obtained pLVX-CMV-EGFP-IRES-Neo (pLVX-EGFP) and pLVX-CMV-PI4Kα-CD-EGFP-IRES-Neo (pLVX-PI4Kα-CD) were also verified by DNA sequencing.

### Lentivirus assembly and lentiviral transfection

HEK-293T cells were used for lentiviral production of the above constructed plasmids pLVX-EGFP and pLVX-PI4Kα-CD *via* liposome-mediated transfection. The packaging vectors were psPAX2 (Addgene: 12260, encoding Gag and Pol) and pMD2.G (Addgene: 12259, encoding VSV-G). Briefly, 300 μl Opti-MEM and 13 μl lipo3000 were mixed and prepared, as well as 300 μl Opti-MEM, 13 μl p3000, 5 μg psPAX2, 2.5 μg pMD2.G and 6 μg pLVX-EGFP/pLVX-PI4Kα-CD mixed and prepared, and then they were mixed in an Eppendorf tube for 5 min followed by adding the mixture into HEK-293T cells cultured in a penicillin/streptomycin-free medium with about 70% confluence in 3.5 cm culture dishes. After 48 h, the lentivirus was harvested and filtered for GBM cells infection plus polybrene. The EGFP^+^ cells were used for measuring the lentiviral infective efficiency. G418 was used for screening the stably transfected cells.

### Specific lentiviral shRNA-mediated knockdown of PI4Kα

For knockdown of PI4Kα by lentivirus-mediated shRNA, two target sequences were designed and provided by Obio Technology (Shanghai) Corp, Ltd for the construction of shPI4Kα lentiviral plasmids and lentivirus assembly. The target sequences were as follows: #1: 5′-CAAGGCTGGATCAACACATAC-3′, #2: 5′-GCGTCTCATCACATGGTACAA-3′. Short hairpin oligonucleotides were designed and then cloned into the empty lentiviral vector pCLenti-U6-shRNA-EF1-EGFP-P2A-Luci2-F2A-Puro-WPRE for lentivirus packaging. The assembled lentivirus was diluted to infect exponentially growing GBM cells at a multiplicity of infection (MOI) of 10 plus polybrene. The infectivity could be estimated by the percentage of EGFP^+^ cells. Puromycin was used for selectively screening the stable transfected cells. The PI4Kα knockdown efficiency was confirmed by western blotting.

### Cell Counting Kit-8 assay and drug treatment

Cell viability was measured by the Cell-Counting Kit 8 assay (CCK-8; Vazyme Biotech Co, Ltd) according to the manufacturer’s instructions. Briefly, 2, 000 cells per well were seeded in 96-well plates, and then cultured with medium containing 2% FBS until the cells adhered to the plate. Finally, CCK-8 solution was added into the wells for reaction and a microplate reader (Varioskan Flash, Thermo Scientific) was used to read the optical density of cells at 450 nm.

XMU-MP-1 (MCE, HY-100526) was dissolved in DMSO and treated with U251 cells at concentrations of 0, 25, 50, and 100 nM for 24 h.

### Cell-colony formation assay

500 cells/well were seeded in a 6-well plate and then cultured for 7 days. After discarding the culture medium, 4% paraformaldehyde (PFA) was added to fix the cell colonies and 0.1% crystal violet was used to stain them, then the number of colonies was counted.

### Wound healing assay

By using a 12-well plate, cells were seeded and cultured until they reached 90% confluence followed by scratching the cell monolayer with a pipette tip to create a wound. The wounds were imaged immediately as the migration initiation (0 h). Then the cells were cultured in medium containing 1% FBS and the wounds were captured at indicated times, 24 h and 48 h, respectively. The wound areas were calculated by using Image J and then the wound closure ratio was calculated.

### Transwell assay

2 × 10^4^ cells in 200 μl FBS-free medium were seeded into the upper chamber of the Transwell chamber (8 μm pore size; Millipore) whereas 500 μl of medium containing 10% FBS was added to the lower chamber. The migrated cells were then fixed with 4% PFA and followed by staining with 0.1% crystal violet. Images were captured under a Nikon inverted light microscope, and the number of migrated cells was counted.

### Western blotting

Cells or tissues were lysed in RIPA lysis buffer containing protease inhibitor cocktail and PMSF. The samples were ultrasonicated followed by placing on ice for 30 min before centrifugation at 14, 000*g* for 15 min at 4 °C. The supernatant was collected and boiled in Laemmli buffer and then separated by SDS-PAGE and transferred to a PVDF membrane. After blocking with 5% skim milk in TBST, the membrane was incubated with primary antibodies at 4 °C overnight. Goat anti-rabbit/mouse IgG HRP-conjugated secondary antibodies (1:10000) from Abcam were used the following day. The primary antibodies used in present study were listed as follows: anti-PI4Kα (#4902s, Cell Signaling Technology (CST), 1:1000), anti-GAPDH (ET1601–4, Huabio, 1:5000), anti-β-Tubulin (#ET1602–4, HuaBio, 1:5000), anti-YAP (#14074, CST, 1:1000), anti-p-YAP (S127) (#4911, CST, 1:1000), anti-p-PI3K (#4228, CST, 1:1000), anti-PI3K (1608–70, HuaBio, 1:1000), anti-Akt(#2920, CST, 1:1000), anti-p-Akt (#13038, CST, 1:1000) and anti-Lamin B1 (#16048, Abcam, 1:5000).

For analyzing the expression of YAP in the nucleus and cytoplasm, a cytoplasmic/nuclear separation kit (EX2650, Solarbio Co, Ltd) was used for separating the nucleus and cytoplasm, followed by western blotting detection.

### Immunofluorescence assay

Cells seeded on poly-L-lysine-pretreated coverslips were fixed with 4% PFA for 30 min and then permeabilized with 0.1% Triton X-100 in PBS for 10 min. After blocking in 5% BSA in PBS at room temperature for 1 h, the cells were incubated with primary antibodies at 4 °C overnight. The primary antibodies used in this study were as follows: anti-PI4Kα (#4902s, CST, 1: 500), anti-phospho-Histone H3 (Ser10) (PH3) (ab14955, Abcam, 1: 500), and anti-YAP (#14074, CST, 1: 200). Then the fluorescent secondary antibodies used was Alexa Fluor 546 goat anti-mouse IgG (H+L) (A11030, Invitrogen, 1: 1000) or Goat anti-rabbit IgG (H+L) Alexa Fluor Plus 546 (A11035, Invitrogen, 1:1000) or Goat anti-rabbit IgG (H+L) Alexa Fluor Plus 647 (A0468, Beyotime, 1:1000) diluted in 5% BSA solution and incubated for 1 h at room temperature in a light-shielded condition. After staining with DAPI (C0065, Solarbio, 1:1000) at room temperature for 10 min followed by sealing with nail polish, images were captured by Olympus SLIDEVIEW VS200 or Nikon A1R MP microscope, and then processed using Adobe Photoshop CS 10.0 software.

### Immunohistochemistry assay

Frozen sections were firstly permeabilized and blocked with 0.3% Triton X-100 in 5% BSA solution at room temperature for 1 h. After washing with 1×PBS, the sections were then incubated with the primary, anti-YAP (#14074, CST, 1:200) and anti-Ki67(#A23722, ABclonal, 1:200) antibodies at 4 °C overnight. The sections were incubated with an enzyme-labeled goat anti-rabbit IgG polymer on the following day at room temperature for 1 h. Then, diaminobenzidine was used to detect the signal followed by hematoxylin counterstaining. Subsequently, the sections were serially dehydrated with 70%, 80%, 95%, and 100% ethanol before immersing in xylene and mounted with neutral resin. Finally, Olympus SLIDEVIEW VS200 microscope was used to capture images.

### Murine orthotopic GBM model

To establish an intracranial mouse GBM model, murine GBM cell line GL261 was suspended in PBS and implanted 1 × 10^5^ cells (1 μl) into the right striatum (+0.6 mm A/P, −1.8 mm M/L, and −3.98 mm D/V) of C57BL/6 mice after they were anaesthetized by using a stereotaxic alignment system. After 3 weeks, mice were perfused and sacrificed for the following assays.

### Hematoxylin-eosin (HE) staining

Frozen sections were stained with hematoxylin followed by washing with double-distilled water, and then stained with eosin solution. The sections were then serially dehydrated with 70%, 80%, 95%, and 100% ethanol before processing with xylene. Neutral resin was used for sealing the sections. Images were captured using Olympus SLIDEVIEW VS200 microscope.

### Quantification and statistical analysis

The statistical significance of the difference between groups was assessed by Student's *t* test, one-way ANOVA or two-way ANOVA. Data were presented as the mean ± SD and *p < 0.05* was considered to indicate a statistically significant difference. GraphPad Prism software version 8.0 was used to perform the statistical analysis.

## Data availability

Data to support this study were available under request.

## Supporting information

This article contains supporting information.

## Conflict of interest

The authors declare that they have no conflicts of interest with the contents of this article.
