# Supplementary material for: Phosphatidylinositol 4-kinase α suppresses glioblastoma progression by inactivating YAP and PI3K/Akt signaling
Source: J Biol Chem. 2026 May 6;302(6):113110. doi: 10.1016/j.jbc.2026.113110 (PMC13254591; doi:10.1016/j.jbc.2026.113110)
Supplement: Supplementary Material [file mmc1.docx]

**Supplementary Material Figure Legends**

**Figure S1. Overexpression of PI4Kα inhibited the growth of C6 cells *in vitro*.**

**(A)** Western blotting was performed to detect the expression of PI4Kα in C6 cells: the upper arrow indicated endogenous full-length PI4Kα and the lower arrow indicated the overexpressed PI4Kα-CD-EGFP. **(B)** Immunofluorescence staining of PI4Kα in control and PI4Kα-CD-overexpressing C6 cells. Scale bar: 20 µm. **(C)** Quantitative analysis of PI4Kα expression as shown in **(B)** (n = 20, *t*-test, ^*^*p*<0.05). **(D)** CCK-8 assay detected the cell viability of control and PI4Kα-CD-overexpressing C6 cells (n = 6, two-way ANOVA, ^*^*p*<0.05, ^**^*p*<0.01). **(E)** Immunofluorescence staining of PH3 in control and PI4Kα-CD-overexpressing C6 cells. Scale bar: 20 µm. **(F)** Quantitative analysis of the percentage of PH3^+^EGFP^+^/EGFP^+^ cells as shown in **(E)** (n = 6, *t*-test, ^**^*p*<0.01). **(G)** Cell colony formation assay was performed in control and PI4Kα-CD-overexpressing C6 cells. Scale bar: 5 mm. **(H)** Quantitative analysis of the number of cell colonies as shown in **(G)** (n = 3, *t*-test, ^**^*p*<0.01). **(I)** Wound healing assay showing the migration of C6 cells with or without PI4Kα overexpression at 24 h and 48 h after scratch. Scale bar: 200 µm. **(J)** Quantitative analysis of the percentage of wound closure as shown in **(I)** (n = 15, two-way ANOVA, ^**^*p*<0.01). **(K)** Transwell assay showing the migrated C6 cells with or without PI4Kα overexpression at 24 h. Scale bar: 50 μm. **(L)** Quantitative analysis of the number of migrated cells as shown in **(K)** (n = 9, *t*-test, ^**^*p*<0.01). Data were represented as mean ± SD.

**Figure S2. Activation of YAP by 50 nM XMU‐MP‐1 increased the cell viability of U251 cells.**

**(A)** CCK-8 assay detected the cell viability of U251 cells treated with 0, 25, 50, and 100 nM XMU-MP-1 for 24 h (n = 6, one-way ANOVA, ^**^*p*<0.01). Data were represented as mean ± SD.
