# Supplementary figures and images for: Phosphatidylinositol 4-kinase α suppresses glioblastoma progression by inactivating YAP and PI3K/Akt signaling
Source: J Biol Chem. 2026 May 6;302(6):113110. doi: 10.1016/j.jbc.2026.113110 (PMC13254591; doi:10.1016/j.jbc.2026.113110)

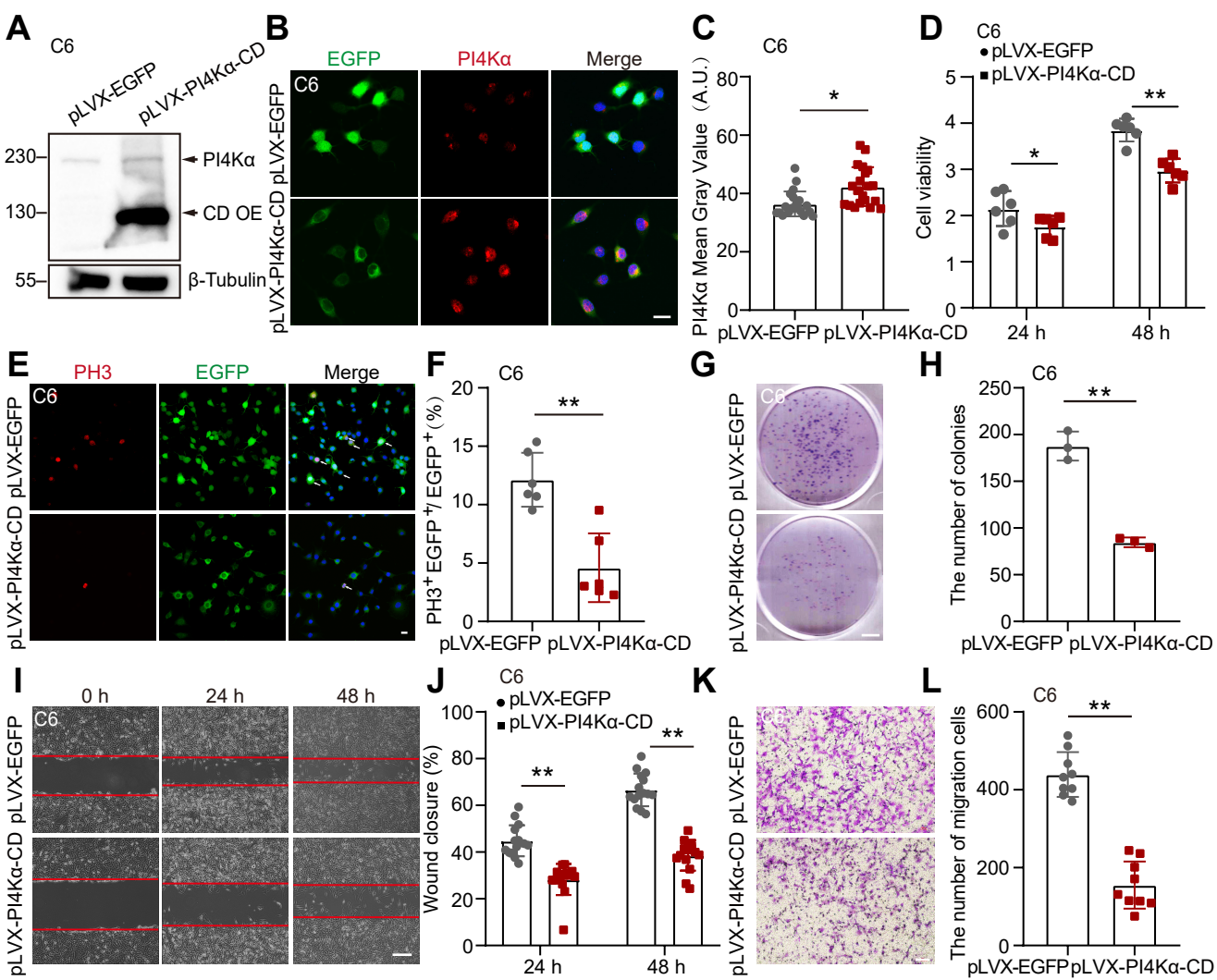

Supplement: Figure S1 [file mmc2.pdf]

**A**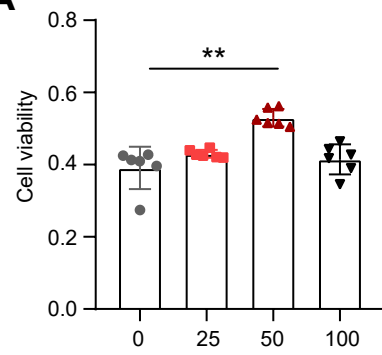

Supplement: Figure S2 [file mmc3.pdf]
